# Personal factors affecting medical professionalism: a qualitative study in Iran

**DOI:** 10.18502/jmehm.v13i3.2842

**Published:** 2020-05-05

**Authors:** Fateme Alipour, Zahra Shahvari, Fariba Asghari, Shahram Samadi, Homayoun Amini

**Affiliations:** 1 Associate Professor, Eye Research Center, Farabi Eye Hospital, Tehran University of Medical Sciences, Tehran, Iran.; 2 Assistant Professor, Department of Nursing and Midwifery, Islamic Azad University of Ghachsaran, Ghachsaran, Iran.; 3 Associate Professor, Medical Ethics and History of Medicine Research Center, Tehran University of Medical Sciences, Tehran, Iran.; 4 Associate Professor, Department of Anesthesia and Intensive Care, School of Medicine, Tehran University of Medical Sciences, Tehran, Iran.; 5 Professor, Department of Psychiatry, School of Medicine, Tehran University of Medical Sciences, Tehran, Iran.

**Keywords:** Medical professionalism, Clinical setting, Qualitative research, Professional behavior, Personal factors

## Abstract

Professional behavior with patients and interactions with colleagues, the institution and professional bodies are influenced by many factors. The purpose of this manuscript is to clarify those personal factors affecting medical professionalism in clinical settings affiliated with Tehran University of Medical Sciences.

For this purpose, a qualitative study was carried out. One hundred and eighty-two participants were recruited through purposive sampling of clinical staff, physicians, and medical students in Tehran. Data were collected through 22 focus group discussions, and conventional content analysis was used to analyze the data.

The results were reported in five categories to present the participants’ views. Categories were extracted from 103 codes and consisted of 1) people's belief in professionalism, 2) personality traits, 3) problems in family, 4) mental or physical health status, and 5) communication skills.

The results showed that despite the facilitator roles of some personal factors, others act as barriers to professional behaviors. In order to control their impact, it is crucial to pay attention to them at the time of student/staff selection. Strengthening support systems in the organization is also essential for decreasing the effect of family problems or physical and mental health problems.

## Introduction

Medical professionalism refers to attitudes, behaviors and relationships (1) that medical professionals should adhere to, in order to be trusted by the society. While trust is a key component of medical professionalism (2), there is some evidence that social trust in medical professionals is decreasing (2). Adhering to the rules of professionalism could improve social trust and quality of care in clinical settings. Commitment to professional behavior in clinical settings is a well-accepted principle categorized under patients' and colleagues' rights, and is considered the responsibility of clinical staff at any level. However, professional behavior toward patients and also interactions with colleagues, institutions and professional bodies are influenced by many factors (3).

Some of these factors act as barriers and challenge professional behavior. Barriers refer to those conditions that make commitment to professional values difficult for medical staff (3).

In facing barriers to professionalism, those who are not trained or lack sufficient experience may show lapses. Attempting to remove these barriers leads an organization to create a professional climate (4). Efforts to recognize and remove or lessen these barriers seem necessary, and so does increasing knowledge in medical professionals.

Some researchers recognized certain aspects of factors that affect professionalism, for instance time constraints, workload, and difficulties in interacting with challenging patients (5), and some identified further factors (6). 

Among the factors affecting professionalism, personal factors have not received sufficient attention; despite their importance, their aspects have not all been evaluated and very few articles have attempted to assess them. However, personal factors have been noted as being significant in predicting professional learning activities (7).

Some research articles have pointed to personal factors indirectly, for example “Physicians’ Knowledge, Attitude and Behavior” (6) or "individual culture" (8). The role of personal factors in adhering to professional behavior has been discussed more directly in some articles, but these are either general (9) or based on review articles (10). Researchers blame some personal factors such as personality traits and lack of communication skills for threatening fundamental professionalism elements such as empathy. They have categorized the personal factors that can potentially impact professional behavior in 3 groups: 1) personal well-being, including quality of life, balance between personal and professional life, burnout, depression and stress; 2) personality traits such as motivation, work ethics, integrity; and 3) interpersonal qualities and skills such as communication skills, compassion, cynicism and detachment (10).

Like any other capability, professionalism is a context-based concept (11) as are its related factors. It seems reasonable to perform qualitative research in order to identify the challenges to professionalism. We evaluated these challenges in the clinical settings affiliated with Tehran University of Medical Sciences (TUMS), and since they were considerable and diverse, we had to report each category separately; one of these categories was personal factors. As managing patients in clinical settings is a team effort involving different levels of care providers, we decided to include all levels of clinical staff in our study, although the focus group sessions were held separately for each group (i.e., attending staff, nurses and trainees). In our view, personal factors are rooted in personal character and attitude and develop throughout life. They start from early childhood or are the result of difficulties in ongoing personal life, and lead people toward professional or unprofessional behaviors. The purpose of this manuscript is to clarify those personal factors affecting medical professionalism in clinical settings.

## Methods

This study was part of an extended qualitative research project on identifying barriers to maintaining professional behaviors in clinical environments using focus group discussions (FGD). 

***Ethical Considerations***

The protocol of the study was approved by the Ethics Committee of Tehran University of Medical Sciences. There were no requirements for participation in the research. Accepting an invitation to attend the focus group sessions was considered as consent to participate in the study. The purpose of research was introduced at the beginning of each session and the participants were assured that the discussions would be kept confidential and only the overall results would be reported. However, they were free to withdraw at any time.The data were collected during 22 focus group discussions (FGDs) with 182 faculty members, residents, interns, nurses, midwives, and other clinical staff. Fourteen sessions were held with non-physician staff, 6 sessions with faculty members and residents, and 2 sessions with interns. Numbers of sessions were determined based on the total number of each group (i.e., attending staff, nurses and trainees) in the university and hospitals, considering the mean accepted invitation for attending in the sessions.

Subjects were invited by faculty (faculty members), student agents (residents and interns), and the Nursing Bureau of the university (nurses), considering their age, sex, and work experience. Response rate was 70% for faculty members, 90% for residents and interns, and 95% for nurses. Inclusion criterion was education or occupation in one of the 13 hospitals affiliated with TUMS, and exclusion criterion was unwillingness to participate in the study. The participants were selected using selective sampling and according to the aims of the research. To ensure all themes were identified, attempts were made to include participants with maximum diversity in terms of age, work experience, work location, grade and field of study. 

The sessions were conducted by 2 of the authors (Shahvari Z. and Alipour A.) in all 13 hospitals affiliated with TUMS for maximizing rate of participation. The mean number of participants in each session was 12. FGDs ranged in length from 90 to 120 minutes (Mean = 105.71 minutes). Data were collected between October 2015 and March 2016. In order to inform the participants about codes of ethics, they were provided with a list of codes of professional conduct based on TUMS codes (12) at the beginning of the sessions. Participants were asked to review the items and express their comments regarding barriers to observance of each item or overall barriers to maintaining a high standard of professional behavior. After each session, the related audio files were transcribed, and the researchers gained an overall sense of each session through frequent readouts of the transcripts. As the next step, the transcripts were coded. 

***Data Analysis and Veriﬁcation***

All FGDs were audio recorded, transcribed verbatim, and checked for accuracy. The data were analyzed using conventional content analysis and version 10 of the MAXQDA software. Data analysis was begun during the data collection phase, and after each FGD, the notes were written down. At the end of each session, two of the authors (Shahvari Z. and Alipour A.) listened to all FGD recordings carefully. The important concepts were recognized and transcribed, and all the transcriptions were re-checked for accuracy. The coded document was discussed by the research team again and revisions were made based on unclear codes. For data categorization. Codes were defined based on the information obtained from the first interviews and revised and completed through reading all the transcripts (13). Categories emerged by inductive reasoning via careful inspection and continuous data comparison (14). To establish trustworthiness of the ﬁndings, we tried to win the trust of the participants by providing a safe environment. No relevant data were excluded or irrelevant data included during data analysis. In addition, the research team tried to increase the validity of the data through long-term engagement and data immersion. The transcripts and codes were verified in group sessions held by the research team. The researchers reanalyzed the corrected transcripts and modified discrepancies in code extraction.

## Results

We reached 103 codes around the personal factors affecting medical professionalism (Table 1). 

**Table 1:** Personal factors affecting professional behaviors

Codes	Sub-Categories	Categories	
**Different families and different morals** **What they have learned from family** **Not believing in honesty in family at all**	• Familial background	Clinical staff’s belief in professionalism	**Personal factors**
**Unwillingness to take part in teamwork ** **Justification of unethical behavior in society** **Misuse of others’ professional behaviors**	• Society-based norms		
**Being cautious** **Being introverted** **Being influential** **Lack of respect for older generation** **Caring about own comfort** **Irresponsibility** **Different values** **Losing the spirit of patience** **Having a different definition of work ethic** **Being self-centered**	• Special traits	Personality traits	
	• Different Millennium Generation Features		
**Facing problems in family life** **Paying loans**	• Common problem	Family problems outside the workplace	
**The kids getting sick** **Facing trouble in life**	• Sudden events		
**Having rheumatologic problems** **Being tempted to resign** **Suffering from various ailments**	• Poor physical health	Mental or physical health status	
**Being aggressive** **Insulting a colleague**	• Poor mental health		
**Interacting well with each visitor** **Calming the atmosphere**	• Good communication skills	Communication skills	
**Being sulky even with each other** **Not knowing how to talk to patients**	• Poor communication skills		

**Figure 1:**
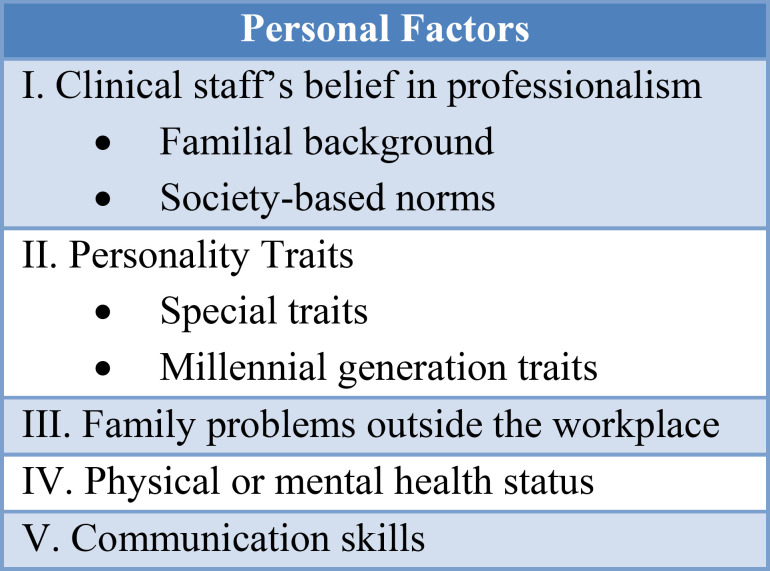
Personal factors affecting professional behaviors

Results are organized according to five categories that clarify those personal factors, affecting professional behavior in clinical settings: 1) clinical staff’s belief in professionalism, 2) personality traits, 3) problems in family, 4) mental or physical health status, and 5) communication skills. An overview of the results is presented in Figure 1.

***I. Clinical Staff’s Belief in Professionalism***

This subcategory refers to those causes of professional misconduct that are related to personal beliefs. Beliefs could be categorized as rooted in “familial background” and/or “society-based norms”.

*-      Familial background*

This category refers to the norms derived from beliefs that originate in the family.

One of the residents said, “*Different families have different morals. People may have been raised in families with lower moral and ethical standards. Not all people are born in moral families. Some people do not believe in honesty at all… whenever a situation is not going well, they turn their back on their own immoral nature”*. Another resident said, “*Personal character should not be neglected.* *Some people have good character and some do not, some people adhere to professional behavior even when they are under high pressure, and some just act professionally under normal situations. Nothing can be done; this is based on their past and what they have learned from family or school*.” 

*-     Society-based norms*

These refer to the norms rooted in the society, including the educational and work environments that medical professionals are part of before entering into clinical practice. Our participants believed “unwillingness to take part in teamwork”, “justification of unethical behavior in society”, and “misuse of others’ professional behaviors” were some negative norms based in the society. Also, “Lack of moral attitude in the society” was considered as an important barrier to professionalism. In this regard one of the residents asked, "*Why should clinical staff behave ethically while other people in the society do not?*”

Behaviors such as lack of adherence to honesty, failure to respect others, egoistic interest-seeking behaviors, and the spread of disrespect and distrust in the society were some of the negative social behaviors that the participants mentioned. In their opinion, medical staff is affected by these trends in the society. Participants pointed out the distrust in interactions between clinical staff and patients, and physicians, patients and staff-managers. In their view, when teamwork is not appreciated in the society, people are not interested in it and will not make an effort to promote it. Participants were also concerned about others’ misinterpretation of their professional behaviors. One of the nurses said, “*If I respect the ward worker - who is much older than me - he won’t do his job perfectly….*”

***II. Personality Traits***

Participants in this study believed that personality traits are among personal factors that push people toward or away from professional behavior. Some participants believed that reserved or introverted people have difficulty in effective communication with patients. One of the nurses commented, “*I have seen many knowledgeable, talented professors who are so immersed in the books that they do not even hear others greeting them! So they have the same behavior with patients and colleagues*”. One resident said, *“In the last flu outbreak, even when we stated that in our opinion as infectious disease subspecialists, there is no need for isolating the patients and observing them from a safe distance and wearing a surgical mask would suffice, many of the nurses and interns avoided approaching the affected patients.*” 

The role of impressive traits as a positive factor was also emphasized. One of the interns said, “*Some people are different from others. They affect their environment. The environment is the same for all of us, but some people always think about patients as their very first priority. We don’t have the right to behave unprofessionally in less than optimal situations, and there are some people in this environment that control the circumstances and make them better.*”

In this regard, a difference was noted between the millennial generation and the older generation. Many of the medical students and clinical staff belong to the millennial generation. They have different values and performance. Losing the spirit of patience and tolerance, having a different definition of work ethic and work conscience, and being self-centered were mentioned as a major difference that may act as a barrier to professional behavior. One of the nurses said, “*The new generation cares more about their own comfort than anything else. They like to earn the highest salary with the least effort… they do not like to bother themselves to help their colleague….*”

***III. Family Problems outside the Workplace***

Anyone may face problems in their family life that are unrelated to the work environment. These problems could affect people’s performance, including their interactions with others. One of the participants said, "*Sometimes a problem in the family causes an individual to be aggressive. We all have families; (for example) our child may become sick…. When someone has trouble in his or her life, she or he certainly cannot offer full service to others.*”

***IV. Physical or Mental Health Status***

From our participants’ point of view, physical or mental health status will affect one’s professional behavior. One of the nurses said, “*Some employees have poor physical health that makes them unfit to work in the clinical setting, and some have poor mental health. There are people who are so aggressive; they insult a colleague over a small issue, while they seem to be so polite and advise others to be calm and polite!* *However, some have good mental health and don’t have any communication skill problems.*”

Work in the hospitals may also exacerbate some physical and mental problems. One of the nurses said,* “Even a very resistant person cannot tolerate the situation for more than 10 years. After ten years you will end up suffering from various ailments. I myself have rheumatologic problems, sometimes I'm tempted to resign.”*

***V. Communication Skills***

Some participants believed that poor or rich communication skills are affected by effective interaction with patients. One nurse said,* “Some employees have good communication skills* *and interact well with each visitor,* *some are sulky even with each other.”*

The excuse of some participants for failure to communicate effectively with patients was high disparity at a socio-cultural level. One nurse said, *“In our workplace, the cultural and social level is low. One does not know how to talk to patients so they won’t be angry.”*

The participants believed that lack of communication skills also affects managers’ and staff’s interactions. One of the nurses said, *“If I protest to my manager that my salary is low, he does not pay attention. He just wants us to work; he does not even sympathize with me because he cannot understand me.”*

## Discussion

The results of this study confirm and extend previous findings (10, 15). 

***Clinical Staff’s Belief in Professionalism***

In our participants’ view “belief in professionalism” is a significant factor for professional behavior. It is rooted in “familial background” and “society-based norms". In stressful settings, people’s moral beliefs will make them behave professionally, and those who do not believe in the necessity of observing their own professional behavior will follow the dominant atmosphere (4). “Low conviction” (16), “negative attitudes toward criteria for medical ethics” and “no belief in compliance with ethical codes” were mentioned as barriers to professionalism commitments (17). Thus, it seems reasonable to pay attention to professional attitude when choosing people for studying or working in clinical majors/environments. 

Overcoming problems caused by the norms stemming from the society requires inter-institutional collaboration. One example identified in this study was teamwork problems. Our participants believed this was rooted in society-based norms. Although many reports emphasize the importance of teamwork in clinical settings (18, 19), and insist that professionalism truly is a team effort (4), teamwork in Iran suffers from numerous weaknesses. As Kalantari et al. suggested, “To improve teamwork and increase quality of behaviors, utilization of educational solutions can be effective” (20). However, managers need to pay more attention to the personnel’s ability to work as team members at the time of recruitment. 

Rectification of all erroneous society-based norms through involvement of the concerned organizations (such as the Ministry of Education and other cultural bodies) is also recommended. In addition, the role of education should not be neglected, both at the level of academic curriculum and in continuing education. 

"Justification for unethical behavior" is also a problem at the society level. Many societies face a decline in moral values, and since clinical staff members come from the same community, they may also be affected. Therefore, raising personnel's moral awareness by organizing regular educational events such as in-depth ethical/professional case discussions in hospitals may cause resistance to wrong social norms. "My colleagues take advantage of my professional behavior" was one issue mentioned by some participants, while others believed that it could not be generalized and was a kind of objection to workload or "justification for unethical behavior". Since professionalism is an interactive behavior, this is a result of lack of awareness about professional commitment, or failure to clarify everyone's duties. In case of "justification", interventions that increase the clarification and close supervision of each ethical code have been shown to be effective (21). Moreover, ethical salience decreases justifications by increasing self-awareness (22).

***Personality Traits***

From the perspective of our participants, cautious and introverted characters may negatively affect professional climate, especially in the field of effective communication, and conversely, people with a positive, impressive character will improve professional climate. Some researchers also believe that certain personality traits are related to the attitude of people to work and work atmosphere (9). Based on our findings, being cautious, introverted, or charismatic are among these traits.

In one research, medical students who were extroverted and self-confident had better social performance (23). Introverted and cautious people have difficulty in socializing and this may make behaviors such as empathy and communicating with patients or colleagues difficult. Personal traits hardly ever change, so paying attention to them at the time of student selection and job allocation seems necessary. Unfortunately, though, the only criterion for student selection in any clinical major is currently their achievement in an MCQ exam. For those members of the staff that are already in a job position, consultation, proper task allocation and education may diminish these negative effects. 

In this study, participants talked about the millennial generation. Challenges between different generations seem to be a worldwide problem. Older generations have difficulty in understanding and respecting the millennial generation (24). The new generation of learners sees the world differently and may have a different perception of professionalism. This highlights the need for the teachers to clarify the expectations of students as they interact in classrooms and clinical settings (25). The millennial generation asks for work-life balance. It does not like ambiguity and demands clear, straightforward rules and interactions (26). Each profession must clarify its behavioral standards to make sure the needs of those it serves are addressed (27). In order to improve professional behavior in this generation, institutions need clear instructions, guidelines and processes. 

***Family Problems outside the Workplace & Physical or Mental Health Status***

“Family problems outside the workplace” and “physical or mental health status” were mentioned in this study as factors that affect professional behaviors. In our opinion, such factors may lead to emotional exhaustion, which has been reported as somehow related to participation in professional learning activities (7), and in this study to behaving in a professional way. Professionalism may require accepting some degree of inevitable risks (e.g., dealing with patients who have contagious diseases or personality disorders. At the same time, work-life balance is important and should not be neglected (28). 

Happiness is affected by different factors, particularly by satisfaction with mental health (29). Some professional behaviors, for instance altruism, are directly linked with happiness and health (30). According to Levinson, organizations must decrease mental concerns for health professionals in practicable ways (4). Existing support systems in an organization may decrease the psychological burden of personal problems on individuals.

***Communication Skills***

Our participants mentioned occasions when disrespect from low social level patients led them to act unprofessionally. In some previous studies, “unusual expectations of patients” and “patients' inappropriate behaviors” were mentioned as barriers to professionalism (16, 17, 31). Although challenging patients are considered a barrier to professional climate (5), those with effective communication skills can connect with everyone for treatment purposes, regardless of their social and cultural levels. Since cultural beliefs add to the complexity of health promotion strategies in health care (32), it seems that some clinical staff consider professionalism rules reciprocal and do not think that everyone deserves to be treated professionally! Insufficient training, unawareness of professionalism rules and weak communication skills could be the reasons for this discrimination. The need for support and/or additional communication skills training was also emphasized. However, cultural sensitivity includes knowledge of the preferred communication styles of different cultural groups (33). 

Weakness in communication skills not only affects interaction with patients, but also causes difficulties in interaction with colleagues. Inappropriate behavior among medical staff was mentioned in a study as one of the ethical issues in neonatal intensive care units (34). It should be remembered that inappropriate feedback and insufficient support from managers and colleagues will decrease job satisfaction and moral sensitivity (35 - 37). As Sprangers et al. emphasized, “Egos, lack of confidence, lack of organization, and structural hierarchies hinder relationships and communications” (38). One communication intervention study demonstrated that the style of speech between nurses and residents was mostly neutral, and positive and negative communications were less common (38). We believe that by providing communication skills training for staff, hospitals can support patient-centered care. Likewise, by demonstrating respect and support for clinicians in daily interactions, hospital administrators can model the same behaviors (4).

Our results were somehow different from the available classifications (10).We emphasized “Clinical staff’s belief in professionalism”, although in previous studies, professional attitudes were considered as a personal factor that could predict professional learning activities (7). Furthermore, this study added “family problems outside the workplace” and “mental or physical health status” to the personal well-being subcategory, as well as “cautious, introverted and impressive character” in the personality traits subcategory of Walt and Shanafelt classification. However, more research is needed for detecting other personality features that may also affect the individual's professional behavior.

The researchers were successful in winning the trust of the participants by providing a safe environment for expressing their experiences, and all participants were involved effectively. 

A large sample size from all levels of clinical care providers, and attending all 13 hospitals affiliated with Tehran University of Medical Sciences were the strong points of this study. Although researchers used the strategy of maximum variation, they realize that this study was a qualitative research and may not be generalizable to clinical settings with different cultural backgrounds. Referring a qualitative study to a specific geographical region was one limitation of this study. Moreover, the results were based on participants’ perspective and may be affected by their judgment or mood.

## Conclusion

Different personal factors may affect professionalism in clinical settings. Despite the facilitator roles of some personal factors, some others act as barriers for professionalism. There are some differences in personality traits between the old and the millennial generation. Not all personalities are considered functional in any given major including medicine and its branches, and some of these factors like personality traits hardly ever change. In order to control their impacts, it is essential to pay attention to them at the time of student selection (for nursing, medical or any clinical majors). Also, personality traits should be considered in job allocation at the time of hiring staff. Training staff will control personal norms and beliefs and improve their communication skills, and strengthening support systems in the organization may decrease the psychological burden of personal problems on individuals.

## Notes:

**Citation to this article:**

Alipour F, Shahvari Z, Asghari F, Samadi S, Amini H. Personal factors affecting medical professionalism: a qualitative study in Iran. J Med Ethics Hist Med. 2020; 13: 3
